# Evaluation of porcine epidemic diarrhea virus transmission and the immune response in growing pigs

**DOI:** 10.1186/s13567-015-0180-5

**Published:** 2015-05-06

**Authors:** Kimberly Crawford, Kelly Lager, Laura Miller, Tanja Opriessnig, Priscilla Gerber, Richard Hesse

**Affiliations:** National Animal Disease Center, United States Department of Agriculture-Agricultural Research Services, Ames, IA USA; Veterinary Diagnostic and Production Animal Medicine, Iowa State University College of Veterinary Medicine, Ames, IA USA; The Roslin Institute, University of Edinburgh, Midlothian, UK; Diagnostic Medicine/Pathobiology, College of Veterinary Medicine, Kansas State University, Manhattan, KS USA

## Abstract

Clinical disease associated with porcine epidemic diarrhea virus (PEDV) infection in naïve pigs is well chronicled; however, information on endemic PEDV infection is limited. To characterize chronic PEDV infection, the duration of infectious virus shedding and development of protective immunity was determined. On Day 0 (D0), a growing pig was challenged with PEDV and 13 contacts were commingled. On D7, 9 contact pigs (principal virus group (PG)), were selected, moved to a separate room and commingled with one sentinel pig (S1). This process was repeated weekly with S2, S3 and S4. The PG was PEDV-positive by PCR from D3-11, with some pigs intermittently positive to D42. Pigs S1 and S2 were PEDV-positive within 24 hours of commingling. Antibodies were detected in all PG by D21 and by 7 days post-contact in S1 and S2. Pigs S3 and S4 were PCR and antibody negative following commingling. To evaluate protective immunity, 5 naïve pigs (N) and the PG were challenged (N/C, PG/C) with homologous virus on D49. All N/C pigs were PEDV PCR-positive by D52 with detection out to D62 in 3/5 N/C pigs. All PG/C pigs were PEDV PCR-negative post-challenge. By D63, all N/C seroconverted. Although PEDV RNA was demonstrated in pigs after primary infection until D42, infectious PEDV capable of horizontal transmission to naïve pigs was only shed 14–16 days after infection to age-matched pigs. Homologous re-challenge 49 days post initial PEDV exposure did not result in re-infection of the pigs. This demonstrates potential for an effective PEDV vaccine.

## Introduction

Porcine epidemic diarrhea virus (PEDV) was discovered in 1976 in the feces of young pigs with diarrhea, and subsequently demonstrated to induce diarrhea in pigs [1]. Retrospectively, this virus was determined to be the cause of an enteric disease in feeder/fattening pigs that was first described in England in 1971, and characterized by severe watery diarrhea with low mortality [2]. Although endemic PEDV infections have persisted in Europe until the present, the economic impact of the virus is considered to be minor [3]. PEDV was first detected in Asia in 1982 when the virus was isolated in Japan [4]. Within a few years it was recognized in other Southeast Asian countries. In contrast to Europe, the clinical impact of PEDV in Asia was much higher leading to the commercialization of both killed and attenuated vaccines in the late 1990s [5]. Vaccine use may have led to a reduction in prevalence of the disease; however, in 2010 severe PEDV outbreaks with high morbidity and mortality in suckling piglets were reported in China and were subsequently attributed to vaccine failure against new viral PEDV strains [5-7].

PEDV is a member of the *Coronaviridae* family and is an enveloped, single-stranded, positive-sense RNA virus with a 28 kb genome encoding non-structural proteins and four major structural proteins including spike, envelope, membrane, and nucleocapsid proteins [8]. The main method of PEDV transmission is fecal-oral; however the ability of the virus to aerosolize and be transported over large distances by air is being considered as an additional important route of virus transmission [9].

PEDV was first identified in the United States in April 2013 in sporadic outbreaks of severe diarrhea in young piglets with high mortality [10]. Within one year the disease spread to 31 states and associated with a 5-7% loss in pig production nationwide [11]. The first isolates identified in the United States had over 99% nucleotide identity to a Chinese isolate from the Anhui province suggesting a Chinese origin of infection, but the primary mode of entry into the United States is still under investigation [12,13]. In January 2014, a variant strain of PEDV with genetic evidence of a Chinese origin was identified in the U.S [14]. Although there is physical evidence for contaminated feed as a mode of transmission in a series of Canadian PEDV cases, such evidence does not exist for the initial introduction of PEDV in North America [15]. Swine are susceptible to PEDV infection at all stages of production with mild diarrhea and vomiting in adults, and severe diarrhea in neonatal pigs causing up to 100% mortality in this age group [3].

Although the clinical disease during an acute outbreak in a breeding herd is well chronicled, little information is available on endemic PEDV infection. The goals of this study were to assess PEDV transmission among pigs, evaluate the duration of shedding of infectious virus, and demonstrate protective immunity of nursery-aged pigs.

## Materials and methods

### Experimental design

**Figure 1:**
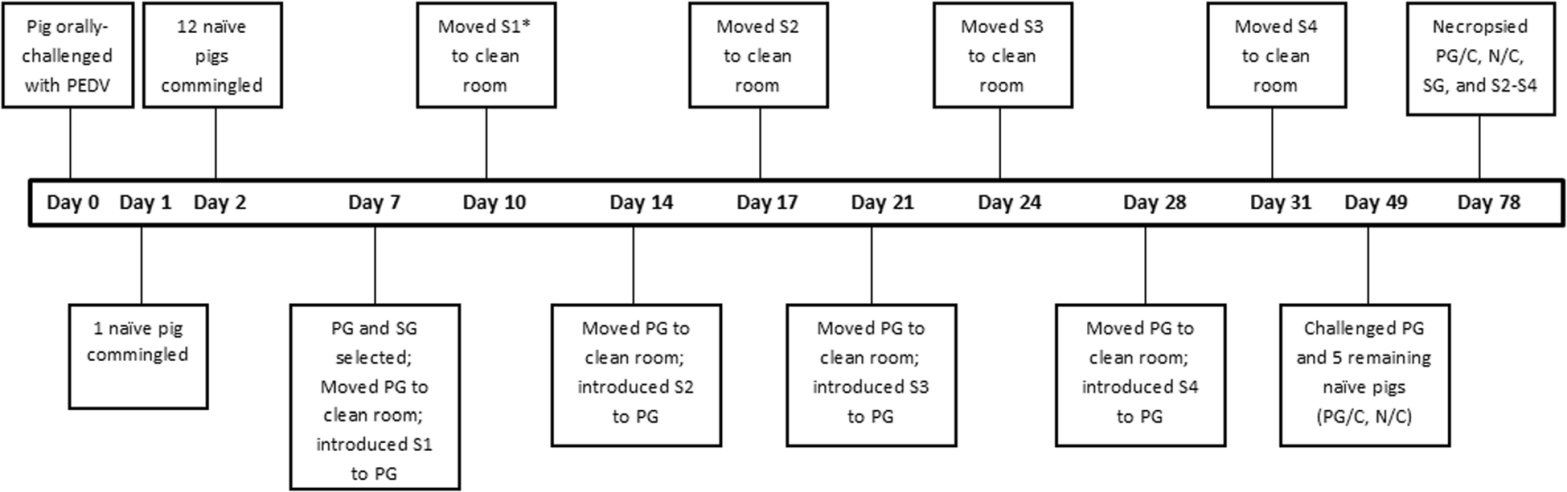
**Experimental timeline of events. Experiment timeline from study initiation (Day 0) to termination (Day 78).** On Day 0, the seeder pig was orally challenged with PEDV. On Days 1 and 2, 1 and 12 contact pigs, respectively were placed with the seeder pig. On Day 7, the Principle Group (PG) consisting of 9 contact pigs, and the Stationary Group (SG) consisting of 4 contact pigs and the seeder pig were selected. On Days 7, 14, 21, and 28, pigs S1, S2, S3, and S4, were commingled with the PG, respectively. On Days 10, 17, 24, and 31, the respective sentinel pig was removed and placed in a separate room. S1 was euthanized and necropsied on Day 30. All other study animals were euthanized and necropsied on Day 78. At Day 49 naïve pigs (N) and PG received PEDV challenge (PG/C: principal virus reservoir group post-challenge. N/C: naïve pigs post-challenge).

Twenty-three, 4-week-old barrows from a PEDV-negative commercial source in Iowa, USA, were randomly assigned a livestock ear tag with a unique number placed in the left ear (Allflex USA, Dallas, TX, USA). A summary of the experimental design and the animal movement can be found in Figure 1. All animals were housed at the National Animal Disease Center, USDA-ARS campus in accordance with Institutional Animal Care and Use Committee protocols (protocol ACUP 2707) following the “*Guide for the Care and Use of Agricultural Animals in Research and Teaching*”. One ABSL2 isolation barn was dedicated to housing non-exposed animals (Barn 1) until moved to isolation Barn 2 for PEDV exposure. On Day (D) 0, a pig was selected from Barn 1 and placed into a room in Barn 2 at which time it was inoculated orally with PEDV and was designated as the “seeder” pig.

On D1 and 2, 1 and 12 naïve contacts, respectively, were commingled with the seeder pig. On D7, 9 pigs were randomly selected from the 13 contact pigs and moved into a new isolation room to serve as the principal virus reservoir group (PG). The seeder pig and 4 remaining contact pigs were designated as the Stationary Virus group (SG) and remained in the same room in Barn 2 throughout the duration of the study. On D7, one naïve sentinel age-matched pig (S1) was commingled with the PG. Seventy-two hours post-contact (D10), S1 was moved to a separate isolation room until necropsy. On D14 the PG pigs were moved into a clean isolation room and pig S2 was commingled until D17 at which time S2 was moved into a clean isolation room until necropsy. This process was repeated on D21 and 28 with pigs S3 and S4. On D49, the five remaining naïve age-matched sentinels (N) in Barn 1 were moved to a clean room in Barn 2. The N and PG were all challenged with PEDV virus and designated as N/C and PG/C, respectively. On D30 (21-days-post contact with PG), S1 was humanely euthanized and necropsied. All other pigs (SG, PG/C, N/C, and S2, S3, S4 pigs) were euthanized on D78. Gross examination was performed on all organ systems and blood was collected for serum.

### Disinfection and maintenance of rooms

Rooms were cleaned daily by removing organic matter with tap water using a high-pressure hose. To prevent cross contamination between groups, room-specific boots, coveralls, and latex gloves were required for each room. Due to animal movement requirements, several rooms in Barn 2 were used multiple times throughout the study; after each group or pig relocation, each room was cleaned and disinfected with a commercial multi-purpose disinfectant (Virkon® S, DuPont, Neogen, Lexington, KY, USA) following this protocol. Pigs and all disposable items were removed from the isolation room leaving an essentially bare concrete room with minimal corners, crevices, etc. If decking or penning was in the room, these items were disassembled as much as possible to allow powerwashing of all surfaces. The room was cleaned with low and high pressure water followed by a soaking with a disinfectant foam. The room was allowed to dry overnight. The second day the air filters were changed and the room was washed and soaked again with disinfectant foam followed by drying overnight. On the following day the room is considered acceptable for pig use. Movement between rooms was in the following order, SG, PG, S1, S2, S3, S4, and after challenge, SG, S2, S3, S4, PG/C and N/C. After moving through each room in sequence, a complete body shower was taken before exiting the barn. No respiratory masks were worn between rooms.

### Clinical observations

All pigs were observed daily for general changes in health including diarrhea, rough hair coat, inappetence, and lethargy.

### Sample collection

Rectal swabs were collected daily from all pigs in Barn 2 using a sterile polyester-tipped applicator (Puritan Medical Products, Guilford, ME) immersed in a 5 mL polystyrene round-bottom tube (BD Falcon, Franklin Lakes, NJ, USA) filled with 2 mL of minimal essential media (MEM). A summary of daily rectal swab collections by group can be found in Tables 1 and 2. The fecal swab samples were frozen at −80 °C until use. Blood was collected from all pigs in Barn 2 on D5, 7, 14, 21, 28, 35, 42, 49, 56, 63, 70, and 77 via jugular venipuncture using a 10 mL syringe with an 18 g X 1 ½” needle (BD Falcon, Franklin Lakes, NJ, USA) then transferred into an 8.5 mL plastic serum separator tube (BD Vacutainer®, 8.5 mL draw; Becton and Dickinson Company, Franklin Lakes, NJ). Blood was centrifuged at 1000 × *g* for 10 min. Following centrifugation, the serum portion of the blood was poured off into 2 mL cryogenic vials (Corning, Corning, NY) and stored at −80 °C until future use.

**Table 1:** Fecal swab PCR results for Stationary Group, Principal Group, and Sentinels from Day 2-42*

	**2**	**3**	**4**	**5**	**6**	**7**	**8**	**9**	**10**	**11**	**12**	**13**	**14**	**15**	**16**	**17**	**18**	**19**	**20**	**21**	**22**	**23**	**24**	**25**	**26**	**27**	**28**	**29**	**30**	**31**	**32**	**33**	**34**	**35**	**40**	**41**	**42**
SG-1	+	+	+	+	+	+	+	+	+	+	+	+	+	+	-	-	-	-	+	-	-	+	+	+	+	-	+	-	+	+	+	+	+	+	-	+	+
SG-2	+	+	+	+	+	+	+	+	+	+	+	-	-	+	-	-	-	-	-	-	-	-	-	-	-	-	-	-	-	-	-	-	+	-	-	-	-
SG-3	-	+	+	+	+	+	+	+	+	+	+	+	+	+	+	-	+	+	+	+	+	+	+	+	-	-	-	-	-	-	-	-	+	-	+	-	-
SG-4	-	+	+	+	+	+	+	+	+	+	+	+	+	+	+	-	-	-	-	-	-	-	-	-	-	-	-	-	-	-	-	-	+	-	-	-	-
SG-5	-	+	+	+	+	+	+	+	+	+	+	+	-	-	-	-	-	-	+	-	-	-	-	-	+	-	-	-	-	-	-	-	+	-	-	-	-
PG-1	-	+	+	+	+	+	+	+	+	+	+	+	+	+	+	+	-	+	+	+	+	+	+	+	-	-	-	-	-	-	-	-	-	-	-	-	-
PG-2	-	+	+	+	+	+	+	+	+	+	+	-	+	+	+	+	+	-	-	-	-	-	-	-	-	-	-	-	-	-	-	-	-	-	-	-	-
PG-3	-	+	+	+	+	+	+	+	+	+	+	+	+	+	+	+	+	+	+	+	+	+	+	+	+	+	-	-	-	-	-	-	-	-	-	-	-
PG-4	-	+	+	+	+	+	+	+	+	+	+	+	+	+	+	+	-	-	-	-	-	-	-	-	-	-	-	-	-	-	-	-	-	-	-	-	-
PG-5	-	+	+	+	+	+	+	+	+	+	+	+	+	+	+	+	-	-	+	-	-	-	-	-	-	-	-	-	-	-	-	-	-	+	-	-	-
PG-6	-	+	+	+	+	+	+	+	+	+	+	+	+	+	+	+	-	-	-	-	-	-	-	-	-	-	-	-	-	-	-	-	-	-	-	-	-
PG-7	-	+	+	+	+	+	+	+	+	+	+	+	+	+	+	+	+	+	+	+	+	-	-	-	-	-	+	+	+	-	-	-	-	-	-	+	+
PG-8	-	+	+	+	+	+	+	+	+	+	+	+	-	-	+	+	-	+	+	+	+	+	-	-	-	-	-	-	-	-	-	-	-	-	-	-	-
PG-9	-	+	+	+	+	+	+	+	+	+	+	+	-	-	+	+	+	+	+	-	-	-	-	-	-	-	-	-	-	-	-	-	-	-	-	-	-
S1						-	+	+	+	+	+	+	+	+	+	+	+	+	+	+	+	+	+	+	+	+	+	+	-								
S2													-	+	+	+	+	+	+	+	+	+	+	+	+	-	-	-	-	+	+	+	+	-	-	-	+
S3																				-	-	-	-	-	-	-	-	-	-	-	-	-	-	-	-	-	-
S4																											-	-	-	-	-	-	-	-	-	-	-

**Table 2:** Fecal swab PCR results for Stationary Group, Principal Group, Sentinels, and Naïve/Challenged group From Day 47–77**

	**47**	**48**	**49**	**50**	**51**	**52**	**53**	**54**	**55**	**56**	**57**	**58**	**59**	**60**	**61**	**62**	**63**	**64**	**65**	**66**	**67**	**68**	**69**	**70**	**71**	**72**	**73**	**74**	**75**	**76**	**77**
SG-1	-	-	-	-	-	-	-	-	-	-	-	-	-	-	-	-	-														
SG-2	-	-	-	-	-	-	-	-	-	-	-	-	-	-	-	-	-														
SG-3	-	-	-	-	-	-	-	-	-	-	-	-	-	-	-	-	-														
SG-4	-	-	-	-	-	-	-	-	-	-	-	-	-	-	-	-	-														
SG-5	-	-	-	-	-	-	-	-	-	-	-	-	-	-	-	-	-														
PG-1	-	-	-	-	-	-	-	-	-	-	-	-	-	-	-	-	-														
PG-2	-	-	-	-	-	-	-	-	-	-	-	-	-	-	-	-	-														
PG-3	-	-	-	-	-	-	-	-	-	-	-	-	-	-	-	-	-														
PG-4	-	-	-	-	-	-	-	-	-	-	-	-	-	-	-	-	-														
PG-5	-	-	-	-	-	-	-	-	-	-	-	-	-	-	-	-	-														
PG-6	-	-	-	-	-	-	-	-	-	-	-	-	-	-	-	-	-														
PG-7	-	-	-	-	-	-	-	-	-	-	-	-	-	-	-	-	-														
PG-8	-	-	-	-	-	-	-	-	-	-	-	-	-	-	-	-	-														
PG-9	-	-	-	-	-	-	-	-	-	-	-	-	-	-	-	-	-														
S2	-	-	-	+	-	-	-	-	-	-	-	-	-	-	-	-	-														
S3	-	-	-	-	-	-	-	-	-	-	-	-	-	-	-	-	-														
S4	-	-	-	-	-	-	-	-	-	-	-	-	-	-	-	-	-														
N/C1	-	-	-	-	**+**	**+**	**+**	**+**	**+**	**+**	**+**	**+**	**+**	-	-	-	-	-	-	-	-	-	-	-	-	-	-	-	-	-	-
N/C2	-	-	-	-	**+**	**+**	**+**	**+**	**+**	**+**	**+**	**+**	**+**	**+**	**+**	**+**	-	-	-	-	-	-	-	-	-	-	-	-	-	-	-
N/C3	-	-	-	-	**+**	**+**	**+**	**+**	**+**	**+**	**+**	**+**	**+**	+	+	+	-	-	-	-	-	-	-	-	-	-	-	-	-	-	-
N/C4	-	-	-	-	**+**	**+**	**+**	**+**	**+**	**+**	**+**	**+**	**+**	+	+	+	-	-	-	-	-	-	-	-	-	-	-	-	-	-	-
N/C5	-	-	-	-	**+**	**+**	**+**	**+**	**+**	**+**	**+**	**+**	**+**	-	-	-	-	-	-	-	-	-	-	-	-	-	-	-	-	-	-

*On Day 0, the seeder pig (SG-1) was orally challenged with PEDV. On Days 1 and 2, 1 and 12 contact pigs, respectively were placed with the seeder pig. On Day 7, the Principal Group (PG) consisting of 9 contact pigs, and the Stationary Group (SG) consisting of 4 contact pigs and the seeder pig were selected. On Days 7, 14, 21, and 28, Sentinel pigs S1, S2, S3, and S4, were commingled with the PG, respectively. On Days 10, 17, 24, and 31, the respective sentinel pig was removed and placed in a separate room. S1 was euthanized and necropsied on Day 30. All other study animals were euthanized and necropsied on Day 78. Samples not collected D36-39. **PG and N/C pigs were orally challenged with CO-13 PEDV on D49. Cycle Threshold of ≤ 35 = positive result (+); Cycle Threshold of ≥ 36 = negative result (−), empty box = sample not collected.

### Inoculum

The PEDV isolate, US/Colorado/2013 (CO-13 PEDV) was supplied by the National Veterinary Services Laboratory, USDA-APHIS, Ames, Iowa (Lot 025 PDV 1303). The virus was propagated on African green monkey kidney (Vero) cells (ATCC, CCL-81) as follows. Vero cells were maintained in MEM supplemented with 10% fetal bovine serum as previously described [16]. Confluent monolayers were infected by decanting the MEM/serum media from a 25 cm^2^ cell culture flask (BD Falcon, Franklin Lakes, NJ, USA), and washing three times with serum-free MEM and 2.5 μg/mL tosyl phenylalanyl chloromethyl ketone (TPCK) trypsin. Cells were inoculated with virus at an MOI of 0.1 in MEM-TPCK and incubated at 38.7 °C. Once cytopathic effect was evident on the cell monolayer, the flask was frozen and thawed twice, decanted into a 15 mL conical centrifuge tube (BD Falcon, Franklin Lakes, NJ, USA) and centrifuged at 1000 × *g* for 10 min. The supernatant was removed, aliquoted, and stored at −80 °C and used for inoculating the seeder pig (CO-13 PEDV stock #1; 1 × 10^5.8^ CCID_50_/mL). A larger stock of virus was prepared in a similar fashion and used to inoculate N/C and PG/C pigs at D49 (CO-13 PEDV stock #2; 1 × 10^6.0^ CCID_50_/mL).

Pigs were physically restrained and the virus inoculum was given orally using a 10 mL syringe (BD Falcon, Franklin Lakes, NJ, USA). The seeder pig was inoculated with a 6 mL volume of stock #1 virus at D0 providing an estimated challenge of 1 × 10^6.6^ CCID_50_ virus. At D49 the N/C and PG/C pigs were given a similar challenge (4 mL volume of stock #2 virus). Aliquots of each challenge virus were frozen at −80 °C for subsequent back-titration which revealed a titer of 1 × 10^5.3^ CCID_50_/mL and 1 × 10^4.5^ CCID_50_/mL for stock virus #1 and #2, respectively.

### RNA extraction and real-time PCR

Each rectal swab sample was thawed and vortexed for 10 s and allowed to sit for 2–5 min to facilitate settling of the fecal debris. Approximately 200 μL of the sample was added to 500 μL of Lysis Binding Solution and mixed using the Thermomixer™ C (Eppendorf, AG, Hamburg, Germany) at 1400 rpm for 5 min at room temperature. The sample was then centrifuged at 13 000 × *g* for 3 min. Clarified lysates were obtained and nucleic acid extractions were performed using the MagMax Pathogen RNA/DNA kit (Life Technologies, Carlsbad, CA, USA) according to the manufacturer’s protocol AM1836-feces (Life Technologies, Carlsbad, CA, USA). The extraction was carried out on the MagMax Express 24 (Life Technologies, Carlsbad, CA, USA). Following extraction, 5 μL of the nucleic acid templates were added to 20 μL of the Ambion® Path ID Multiplex One-Step RT-PCR reaction master mix (Life Technologies, Carlsbad, CA, USA). The PEDV N and S-gene real time RT-PCR reactions were based on recommendation by the University of Minnesota Veterinary Diagnostic Laboratory [17] and were ran in standard mode on a 7500 Fast Real-Time PCR System (Life Technologies, Carlsbad, CA, USA). The thermal cycler protocol started with 10 min at 45 °C, followed by 10 min at 95 °C. The final cycle consisted of 15 s at 95 °C and then 45 s at 60 °C. The final cycle was run for 40 cycles using the FAM detector for PEDV. Positive and negative controls were included on each run. A cycle threshold (Ct) of less than 35 cycles was considered a positive result.

### PEDV Spike 1 protein ELISA

The ELISA test was performed using a previously published method [18]. Briefly, each well of a 96-well microtiter plate (Nalgene Nunc International, Penfield, NY) was coated with 0.44 ng of S1 protein, incubated overnight, and blocked with 1% bovine serum. The 1/100 diluted samples of serum in PBS with 10% goat serum were reacted at 37 °C for 30 min, washed, and incubated with 20 000-fold diluted peroxidase-conjugated goat anti-porcine IgG. Using tetramethylbenzidine-hydrogen peroxide as the substrate, the reaction was visualized for 10 min at room temperature and terminated with 2.5 M sulfuric acid prior to OD measurement at 450 nm. Positive, negative, and blank samples were tested in duplicate on each plate.

## Results

### Clinical assessment

A watery, grey diarrhea was noted in the seeder pig 48 hours-post-inoculation with the cell culture-derived challenge material and lasted for approximately 5 days. Approximately 48–72 hours post-exposure with the seeder pig, the contact pigs showed intermittent signs of mild-moderate diarrhea lasting for approximately 7 days. No other clinical signs of diarrhea were appreciated in the contact pigs throughout the remainder of the study. S1, S3, and S4 showed no signs of diarrhea, while S2 had diarrhea for approximately 1 day 24 hours-post-contact with the PG. Post-challenge, the N/C group showed clinical signs of mild diarrhea beginning approximately 48 hours-post-inoculation that lasted for approximately 5 days with intermittent diarrhea in a few pigs for 3 weeks post-challenge. In contrast, no clinical signs were noted in the PG/C pigs post-challenge. The rectal swab fluids were not tested for other enteric pathogens.

### PEDV RNA detection

On D0 all fecal swabs were negative for PEDV RNA. The seeder pig (SG-1) and contact pigs were PCR positive within 24 hours of inoculation (seeder pig) or contact (13 contact pigs). PCR results for D2-42 fecal swabs are summarized in Table 1. The seeder pig was positive from D1-15, and then intermittently thereafter through D42. The 4 contact pigs that were combined with the seeder pig to form the SG group were all positive from D3-12 and then intermittently through D35. PEDV RNA was detected in all PG pigs from D3-11, and at least 1/9 PG pigs was PCR positive from D3-30. From D30-34, 0/9 PG pigs were PCR positive. Fecal swabs were not collected from the PG pigs from D36-39 but on D35 and 42, 1/9 PG pigs showed a positive result. Each sentinel pig was negative for PEDV RNA at the time of contact with PG. PEDV RNA was detected in pigs S1 and S2 within 1 day of contact with the PG. S1 was positive until necropsy at 21 days post contact. S2 was positive for 11 days post contact and then intermittently afterwards through D34 (20 days post contact). PEDV RNA was not detected in any sample obtained from pigs S3 (commingled with PG on D21) and S4 (commingled with PG on D28).

After re-challenge at D49, all PG/C pigs were PCR negative for the remainder of the study (Table 2). Within three days post challenge (D52), 5/5 N/C pigs were PCR positive and remained so until D59, while 3/5 remained positive until D62.

### PEDV antibody detection

**Figure 2:**
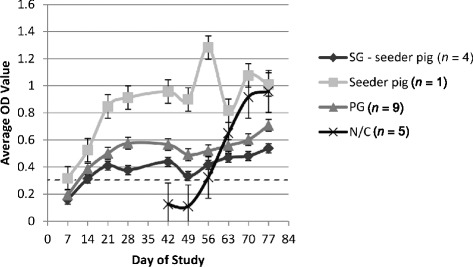
**Average Optical Density (OD) of IgG antibody to PEDV.** Average (OD) of IgG Antibody to PEDV over time in the Stationary Group without seeder pig included (SG - seeder), Seeder pig, Principal Group (PG), and Naïve/Challenge (N/C) group. On D 49, PG and N/C pigs orally challenged with PEDV strain USA/Colorado/2013. Dashed line represents cut-off optical density value of 0.3.

In the PG group, 2/9, 8/9, and 9/9 pigs were positive for PEDV-antibodies by D7, 14, and 21, respectively. In the N/C group, 2/5 pigs were ELISA positive by D56 (7 days post challenge) and all pigs were positive from D63 to D78. S1 and S2 seroconverted by 7 days post-contact and S3 and S4 remained seronegative throughout the remainder of the study. Average ELISA OD readings for the PG, SG, and N/C groups are depicted in Figure 2.

## Discussion

The rapid dissemination of PEDV across the US in months demonstrated the vulnerability of a concentrated, interwoven swine production system to a highly transmissible novel enteric pathogen. This unprecedented spread was dependent on many factors including the duration and magnitude of virus shedding from infected to naïve pigs. The goal of this study was to better understand the role of pig-to-pig transmission in the US PEDV epidemic by developing a model to characterize the host response to infection with PEDV.

In 4-week-old contact pigs, mild-to-moderate diarrhea was observed for approximately one week post exposure to a PEDV experimentally-infected seeder pig. No other clinical disease was observed in the contact pigs. Within 24 hours-post-inoculation or post-exposure, all rectal swabs were PCR positive demonstrating the rapidity of transmission and the potential for pigs to shed infectious virus. In the pigs “naturally” infected by contact, 13/13 pigs were PCR positive for 9 days and most pigs were positive for 2-weeks-post exposure during which time one or more of the pigs shed an infectious dose of virus to pigs S1 and S2. Like the 13 naturally exposed pigs, S1 and S2 became PCR positive within 24 hours of contact, and seroconverted within 2 weeks post-exposure. The speed by which all pigs became PEDV RNA positive and the potential for pigs to shed infectious virus up to 2-weeks-post infection to age-matched pigs, suggests the basic reproduction number or *R*_*0*_ should be high for this virus.

As would be expected for a virus with a high *R*_*0*_ value, the transmissibility of the virus would be enhanced by large numbers of naïve animals concentrated in small areas. In the case of PEDV, an enteric virus that can be shed in feces in large quantities, it is not a surprise that the virus would heavily contaminate transportation systems that would facilitate its spread throughout the US. Moreover, potential spread of the virus through contaminated feed and by air would allow this virus to circumvent most bio-security practices. Collectively, these factors combined to make a “perfect storm” for spread of PEDV in the US.

Based on negative PCR results and lack of specific antibody, pigs S3 and S4 were determined to have remained PEDV negative despite contact with PG pigs that were intermittently PCR positive. The negative status of S3 and S4 indicates the PG pigs did not shed infectious virus in sufficient quantity to infect age-matched pigs (aged 7 and 8 weeks, respectively). It is not known if younger, presumably more susceptible pigs might have become infected under similar conditions. Likewise, we do not know if the infectious character of the virus being shed changes with time, i.e., the rectal swabs are PCR positive but may not contain infectious virus. For this experiment, the PCR results were defined as positive or negative based on a Ct cutoff of 35, as recommended by the University of Minnesota Veterinary Diagnostic Laboratory. Whether or not this positive/negative cutoff correlates to infectious/non-infectious virus is not known.

Protective immunity to homologous challenge was observed in the PG pigs based on the absence of clinical disease following challenge, and the pigs remained PCR negative from D49 through 63. In contrast, following challenge at D49, the 11-week-old N/C pigs developed an intermittent mild diarrhea beginning 2–3 days post-challenge that lasted for several days. Within 48-hours post-challenge, 4/5 pigs were PEDV PCR positive and all 5 were positive from D52-59. The number of positive pigs reduced to 3/5 positive from D60-62.

Field reports describe age differences in clinical PEDV disease ranging from essentially 100% mortality in neonatal pigs to a moderate to mild diarrhea in older pigs and adults that may include vomiting. The minimal infectious dose, and the minimal lethal dose for different ages of swine are not known. Moreover, there could be considerable differences in the pathogenicity of different PEDV isolates, and innate resistance between genetic lines of swine. For the purposes of this paper, we chose to give a uniform dose to the younger (4 weeks-of-age) and older (11 weeks-of-age) pigs realizing that the older pigs might be less affected due to a relatively smaller dose, and the possibility that there may be inherent resistance to infection as pigs mature. Characterizing the relationship between age, challenge dose, and clinical disease is beyond the scope of this study. Although both age groups did become infected following challenge, back titrations of challenge virus suggests the younger pigs received a larger challenge dose than the older pigs (about 6 log_10_ vs. 5.1 log_10_) which limits any interpretation of the potential relationship between age, challenge dose and disease.

In this study, the 4-week-old pigs were more clinically affected when compared to 11-week-old pigs. None of the pigs in this study became clinically dehydrated or succumbed to the infection, and other than showing signs of diarrhea, it would have been difficult to discern any apparent illness if the pigs were housed on slatted floors. We presume this difference is mostly age-related as younger pigs are more susceptible to disease. However, the younger pigs became infected by contact with a seeder pig while the older pigs received a known oronasal challenge of cell culture propagated virus, and it is not known how these different routes of exposure might affect the pig. Although the study was not designed to compare the clinical effect of cell-culture propagated virus challenge vs. natural infection, it is interesting to compare the ELISA OD values for the one seeder and 5 N/C pigs that received an oronasal challenge to the 13 naturally infected pigs (Figure 2). The mean OD of the 6 inoculated pigs was higher than the naturally exposed pigs at each post-inoculation time point (7, 14, 21, and 28 days-post-inoculation). The importance of this trend and whether or not it would be reproducible in subsequent studies is not known. In addition, for the 13 contact pigs, the mean OD value was greater at each post-exposure time point for the PG group when compared to the 4 contact pigs in the SG group. This apparent trend could reflect exposure, albeit a short exposure, to “fresh” virus being shed by S1 and S2 when they were commingled with the PG for 3 days at D7 and 14, respectively. Perhaps this potential exposure affected the development of the humoral antibody response. Under the conditions of this study, an anamnestic humoral immune response was not observed following challenge of the 9 PG pigs. Lack of immunological memory was also observed in a report using the Belgian isolate, CV-777 [19]. Given that the N/C pigs replicated challenge virus and developed specific antibody, we are confident the PG pigs received an infectious homologous challenge and had no detectable replication of homologous challenge virus or rise in humoral antibody titer.

The infection of contact pigs exposed to the seeder pig, and the infection of S1 and S2 pigs demonstrated pig-to-pig transmission of infectious virus. In the case of the seeder and contact pigs, transmission happened quickly since all of the contact pigs were rectal swab positive within 24 h of contact to either the seeder pig, or the seeder pig and first contact pig. These results are similar to a recent report using a different US PEDV strain [20]. Similarly, S1 and S2 pigs were rectal swab positive within 24 h of contact with the PG group. Collectively, these results indicate pigs can rapidly become infected and shed infectious virus for at least 2 weeks. However, this study does not provide insight into the relationship between samples that are deemed positive by PCR and the presence of infectious virus in rectal swab fluids. In the case of the seeder pig infecting the 13 contact pigs, the seeder pig was positive from D1-15 and clearly shed infectious virus on D1 since the first contact pig was PCR positive by D2. Whether the 12 remaining contact pigs became infected by exposure to virus from the seeder pig or the first contact pig is unknown, but all were positive 24 h later on D3. All 9 PG pigs were PCR positive on D7 when S1 was commingled and at least one of these pigs shed infectious virus since S1 was PCR positive by the next day. When S2 was commingled on D14, 7/9 PG pigs were PCR positive and at least one was shedding infectious virus since S2 was positive on D15. At D21 and 28, 4 and 1 of 9 PG pigs were PCR positive, respectively, but none of the pigs shed an infectious dose based on lack of demonstrable infection in S3 and S4. The Negative/Positive status of the rectal swab was based on using the Ct value of 35; negative (>35) or positive (<35). This cutoff value was determined by the original designers at the University of Minnesota Veterinary Diagnostic Laboratory and is routinely used by other diagnostic laboratories performing either the original real-time PCR assay, or a modification of the assay (D. Madson, personal communication). Additional studies are warranted to understand the relationship between Ct value and the presence of infectious virus.

There is justifiable concern by the swine industry on how robust this virus is and what efforts are necessary to eliminate environmental contamination. In this study, there were movements of pigs into rooms that had previously housed PEDV-infected pigs. We did not conduct any environmental sampling of the rooms for PEDV contamination prior to use, thus we do not know what potential contamination might have existed in the room from preceding pigs. Each room was cleaned following the removal of previous pigs according to our standard protocol for cleaning the ABSL-2 isolation rooms. During the acute phase of the experiment it was not possible to assess if infectious virus may have been present in the room upon entry of the pigs because the pigs were already positive. Later in the experiment, we did not find evidence for pigs becoming infected upon movement into a room, i.e., S3 and S4 did not become infected when moved from the PG into separate isolation rooms. In addition, these rooms have been used subsequently for non-PEDV pig studies and no PEDV contamination was detected. Based on these experiences, we believe our routine cleaning protocol was adequate to inactivate PEDV contaminated surfaces.

In general, results from this study agree with recent observations by others that have experimentally infected 3- and 4-week-old pigs with US PEDV isolates resulting in the production of mild to moderate clinical disease [20]. Conversely, prior to this study, the longest duration of fecal shedding of PEDV was reported out to 24 days post-inoculation [20]. We detected intermittent viral shedding by PCR in several pigs up to D42 even though clinical signs diminished approximately 7 days post exposure. To our understanding, this is the longest length of PEDV shedding reported in pigs to date. Asymptomatic shedding of PEDV in pigs introduces a higher level of difficulty in the management of the disease throughout the swine industry.

In summary, this study and the work of others demonstrates how easily pigs become infected with PEDV, and may help explain the rapid transmission of virus recently observed in the US. In addition, pigs shed infectious virus for 2 weeks which would help explain how easily the virus was transmitted among farms. The apparent sterile immunity following primary infection suggests there may be value in a consistent feedback program, and it demonstrates potential for a vaccine to help manage the disease.

## References

[1] Pensaert MB, De Bouck P (1978). A new coronavirus-like particle associated with diarrhea in swine. Arch Virol.

[2] Wood E (1977). An apparently new syndrome of porcine epidemic diarrhoea. Vet Rec.

[3] Saif L, Pensaert MB, Sestak K, Yeo SG, Jung K (2012) From Coronaviruses. In Disease of Swine. 10th edition. Edited by Zimmerman JJ, Karriker LA, Ramirez A, Schwartz KJ, Stevenson GW. Ames, Iowa, USA; Wiley-Blackwell. pp 501–524

[4] Takahashi K, Okada K, Ohshima K (1983). An outbreak of swine diarrhea of a new-type associated with corona-virus like particles in Japan. Nihon Juigaku Zasshi.

[5] Wang XM, Niu BB, Yan H, Gao DS, Yang X, Chen L, Chang HT, Zhao J, Wang CQ (2013). Genetic properties of endemic Chinese porcine epidemic diarrhea virus strains isolated since 2010. Arch Virol.

[6] Sun RQ, Cai RJ, Chen YQ, Liang PS, Chen DK, Song CX (2012). Outbreak of porcine epidemic diarrhea in suckling piglets, China. Emerg Infect Dis.

[7] Li R, Qiao S, Yang Y, Su Y, Zhao P, Zhou E, Zhang G (2014). Phylogenetic analysis of porcine epidemic diarrhea virus (PEDV) field strains in central China based on the ORF3 gene and the main neutralization epitopes. Arch Virol.

[8] Song D, Park B (2012). Porcine epidemic diarrhoea virus: a comprehensive review of molecular epidemiology, diagnosis, and vaccines. Virus Genes.

[9] Alonso C, Goede DP, Morrison RB, Davies PR, Rovira A, Marthaler DG, Torremorell M (2014). Evidence of infectivity of airborne porcine epidemic diarrhea virus and detection of airborne viral RNA at long distances from infected herds. Vet Res.

[10] Stevenson GW, Hoang H, Schwartz KJ, Burrough ER, Sun D, Madson D, Cooper VL, Pillatzki A, Gauger P, Schmitt BJ, Koster LG, Killian ML, Yoon KJ (2013). Emergence of porcine epidemic diarrhea virus in the United States: clinical signs, lesions, and viral genomic sequences. J Vet Diagn Invest.

[11] Novel Swine Enteric Corona Virus Disease Testing Summary Report. [www.aasv.org/pedv/SECoV_weekly_report_140425.pdf]

[12] Huang YW, Dickerman AW, Piñeyro P, Li L, Fang L, Kiehne R, Opriessnig T, Meng XJ (2013). Origin, evolution, and genotyping of emergent porcine epidemic diarrhea virus strains in the United States. MBio.

[13] Chen Q, Li G, Stasko J, Thomas JT, Stensland WR, Pillatzki AE, Gauger PC, Schwartz KJ, Madson D, Yoon KJ, Stevenson GW, Burrough ER, Harmon KM, Main RG, Zhang J (2014). Isolation and characterization of porcine epidemic diarrhea viruses associated with the 2013 disease outbreak among swine in the United States. J Clin Microbiol.

[14] Wang L, Byrum B, Zhang Y (2014). New variant of porcine epidemic diarrhea virus, United States, 2014. Emerg Infect Dis.

[15] Pasick J, Berhane Y, Ojkic D, Maxie G, Embury-Hyatt C, Swekla K, Handel K, Fairles J, Alexanderson S (2014). Investigation into the role of potentially contaminated feed as a source of the first-detected outbreaks of porcine epidemic diarrhea in Canada. Transbound Emerg Dis.

[16] Hoffman M, Wyler R (1988). Propagation of the virus of porcine epidemic diarrhea in cell culture. J Clin Microbiol.

[17] University of Minnesota Diagnostic Laboratory PEDV Rapid Diagnostic Test [http://www.cvm.umn.edu/sdec/prod/groups/cvm/@pub/@cvm/@sdec/documents/content/cvm_content_446628.pdf]

[18] Gerber PF, Gong Q, Huang YW, Wang C, Holtkamp D, Opriessnig T (2014). Detection of antibodies against porcine epidemic diarrhea virus in serum and colostrum by indirect ELISA. Vet J.

[19] De Arriba ML, Carvajal A, Pozo J, Rubio P (2002). Mucosal and systemic isotype-specific antibody responses and protection in conventional pigs exposed to virulent or attenuated porcine epidemic diarrhoea virus. Vet Immunol Immunopathol.

[20] Madson D, Magstadt D, Arruda P, Hoang H, Sun D, Bower L, Bhandari M, Burrough E, Gauger P, Pillatzki A, Stevenson G, Wilberts B, Brodie J, Harmon K, Wang C, Main R, Zhang J, Yoon KJ (2014). Pathogenesis of porcine epidemic diarrhea virus isolate (US/Iowa/18984/2013) in 3-week-old weaned pigs. Vet Microbiol.

